# The receipt of information by family physicians about their patient’s emergency department visits: a record linkage study of electronic medical records to health administrative data

**DOI:** 10.1186/s12875-021-01582-x

**Published:** 2021-11-22

**Authors:** Liisa Jaakkimainen, Hannah Chung, Hong Lu, Bogdan Pinzaru, Elisa Candido

**Affiliations:** 1 Primary Care and Health Systems, ICES, 2075 Bayview Ave, G Wing, Toronto, M4N 3M5 Canada; 2 Department of Family and Community Medicine, University of Toronto, Toronto, Canada; 3 Institute of Health Policy, Management and Evaluation, University of Toronto, Toronto, Canada; 4 Department of Family and Community Medicine, Sunnybrook Health Sciences Centre, Toronto, Canada

**Keywords:** Emergency room visits, Family medicine, Administrative data, Electronic medical records, Information coordination

## Abstract

**Background:**

Canadians are known to be frequent users of emergency department (ED) care. However, the exchange of information from ED visits to family physicians (FPs) is not well known. Our objectives were to determine whether Canadian FPs received information about their patient’s ED visit and the patient characteristics related to the receipt of ED information.

**Methods:**

This study was a descriptive record linkage study of FP Electronic Medical Record (EMR) data linked to health administrative data. Our study cohort included patients who had at least one ED visit in 2010 or 2015 in Ontario, Canada. An ED visit could include a transfer to or from another ED. The receipt of information from an ED note was examined in relation to patient age, sex, neighbourhood income quintiles, rurality and comorbidity.

**Results:**

There were 26,609 patients in 2010 and 50,541 patients in 2015 with at least one ED visit. In 2010, 53.3% of FPs received an ED note for patients having a single ED visit compared to 41.0% in 2015. For patients with multiple ED visits, 58.2% of FPs received an ED note in 2010 compared to 45.7% in 2015. FPs were more likely to receive an ED note for patients not living in low income neighbourhoods, older patients, patients living in small urban areas and for patients having moderate comorbidity. FPs were less likely to receive a note for patients living in rural areas.

**Conclusions:**

Community-based FPs are more likely to get information after an ED visit for their older and sicker patients. However, FPs do not receive any information from EDs for over half their patients. Electronic health record technologies and their adoption by ED providers need to improve the seamless transfer of information about the care provided in EDs to FPs in the community.

## Background

Canadians are known to be frequent users of emergency department (ED) care amongst developed nations [1, 2]. Canadian ED users were ranked number one in an international comparison of 34 countries when they indicated they went to the ED because their family physician (FP) could not provide the care they needed [3]. Only 34% of Canadians said they could get evening or weekend care without going to the ED. [4] However for people who saw their FP regularly for their chronic disease management, ED use was reduced [5–7]. The benefits of improved access to after-hours care have been equivocal, with little difference in ED visits amongst primary care delivery models that offer better after-hours access [8]. Indeed, not having a FP or poor access to a FP may not be the reasons for frequent use of EDs by some people [9, 10]. Regardless of the reasons Canadians go to the ED, it is important to have the information gained during an ED visit provided to a patient’s FP so that acute care is followed up, complications from chronic diseases are better managed and mental health illnesses are supported.

Since the receipt of hospital discharge information by a patient’s FP has been associated with improved chronic disease management and fewer prescribing errors [11–15], recommendations have been made to ensure a hospital discharge note/summary is available to a FP within 48 h of a patient leaving the hospital [16]. With an increasing proportion of ED visits made by patients who are older and more medically complex, there is a need for FPs to have information from their patient’s ED visit [17]. Inadequate follow up after an ED visit is associated with disease complications [18, 19]. Furthermore, improving the coordination of care between ED and primary care could improve the duplication of treatments, opportunities to educate patients on appropriate ED use and better disease management [20]. However, little information exists on whether FPs received information from EDs.

Canadians have universal healthcare where all medically necessary physician visits are provided to residents and paid and managed by provincial government health plans [21]. In Ontario, Canada’s most populous province, FPs are the physician providers of primary care for adults and most children [22]. With the initiation of primary care reform in Ontario in 2003, an increasing proportion of patients are formally enrolled (rostered) to an individual FP for all their primary care [23–25]. In 2017, 88.6% of people in Ontario have a FP, with 82.9% being formally enrolled (rostered) with a FP [26].

The initial focus of Electronic Health Technology (EHT) adoption by Ontario EDs was to support ED clinicians with point-of-care access to investigations (such as laboratory and radiological test results), as concerns about information gaps on patient care existed [27]. In 2014 the provincial digital health strategy included the expansion of an integrated EHT, including improvements with communication and connectivity from EDs [28]. Prior to 2014, the standard method of communication from an ED physician to a FP was a hand-written one-page report provided directly to the patient or FAXed to their FP. Once received by the FP’s office, these ED notes would subsequently be scanned into a FP electronic medical record (EMR). The provincial digital health strategy specifically includes the expansion of a hospital medical record (HMR) system whereby FP EMRs directly receive hospital discharge summaries, notes from ED visits and the results of some radiological investigations [29]. However, there is no data which estimates whether community-based FPs actually receive this information.

In 2019, 86% of Canadian FP practices use EMRs for their clinical care [30]. FP EMRs contain a wealth of information describing care provided to their patients and this data has increasingly been used for research and quality improvement [31, 32], including how well the healthcare system coordinates care across different health care sectors [33].

Therefore, the objectives of this study were to determine whether information from a patient’s ED visit is received by their FP, to examine patient factors related to the receipt of ED information and to see if changes in the receipt of ED information by FPs has occurred between 2010 and 2015.

## Methods

### Study design

We conducted a descriptive analysis on a cohort of patients enrolled with FPs in Ontario, Canada who went to the ED in 2010 and 2015.

### Sources of data

We used information gathered from the Electronic Medical Records Primary Care (EMRPC) database held at ICES, which is comprised of EMRs from a network of community-based FPs across Ontario [34]. EMRPC contains the entire clinical record of FP patients, including their cumulative patient profile, progress notes, consultant notes, radiological and laboratory tests, hospital and emergency discharge notes and referrals. ICES is a ‘prescribed entity’ under Ontario privacy legislation which provides the legal authority to collect individual level health information, as ICES has the policies and procedures in place to protect patient privacy and confidentiality [35]. The health administrative data for this study included ED visits from the Canadian Institute for Health Information National Ambulatory Record System (NACRS) database, physician encounter claims from the Ontario Health Insurance Plan (OHIP) database and patient age and sex held in the Registered Persons Database (RPDB). These datasets were linked using unique encoded identifiers and analyzed at ICES.

### ED study cohort

Our study cohort include EMRPC patients who had at least one NACRS ED visit in 2010 or 2015. EMRPC patients were excluded if they did not have a valid health card number or date of birth, they were not formally enrolled (rostered) to the EMRPC FP or if their ED visit resulted in an admission to hospital.

We used previously standardized methods to define an ED visit from ED health service research in Ontario [36]. An ED visit may include multiple NACRS ED visit claims or records. For example, an ED visit could include an ED visit that involves a transfer to another ED or facility and an ED visit which is a transfer from another ED or facility or a revisit within 28 days. An ED revisit within 28 days with no indication of an ED transfer is not considered the same ED visit and would be considered a separate ED visit. For our ED study cohort, we separated patients having one ED visit versus patients having multiple ED visits.

### ED information in FP EMR notes

All EMRPC specialist consultant notes for each patient were examined starting after the ED visit dates up to 6 months after the end each study cohort. We used a hieratical method to identify ED notes/information in the EMR. First, we identified the EMR ED note using a date which was the same as the ED visit date. For the remaining ED visits, we identified any type of specialist consultant note with the same date as the ED visit date. Finally, for the remaining ED visits we identified any notations in the EMR with terms “Emerg”, “ER”, “Urgent Care” and their misspellings and combinations. These notations included non-note formats such as laboratory and radiological test results.

### Covariates

The covariates in our study included patient age, sex, rurality, neighbourhood income quintiles and comorbidity. Statistics Canada’s postal code conversion files and census data were used to calculate neighbourhood income quintiles [37] and the location of residence as defined by the rurality index of Ontario [38]. The Johns Hopkins ACG® System Aggregated Diagnosis Groups (ADGs) were used to measure patient comorbidity from all health care encounters in the year prior to their ED visit [39]. Validated chronic disease administrative data algorithms were used to identify comorbidities such as a previous history of an acute myocardial infarction (post-AMI), asthma, chronic obstructive pulmonary disorder (COPD), congestive heart failure (CHF), diabetes, hypertension or mental health conditions [40–47].

### Statistical analysis

For 2010 and 2015, we calculated the proportion of ED notes received by FPs for both patients with a single ED visit and those with multiple ED visits. Single ED visit patients were counted as having an ED note if at least one note was received. Multiple ED visit patients were counted as having an ED note if a note from any ED visit was received. We conducted bivariate analyses of patient characteristics between patients having single or multiple ED visits in 2010 and 2015. For 2015, we compared patient characteristics when their FP received and or did not receiving an ED note. A *p* < 0.001 indicated statistical significance. Logistic regression was undertaken to examine the association of patient characteristics on their independent role with the receipt of any ED notes. All analyses were performed using SAS Enterprise Guide version 7.1 (Cary, NC) [48].

## Results

For our primary care patient cohort, there were 26,609 patients in 2010 and 50,541 patients in 2015 with at least one ED visit. In both years, most patients had a single ED visit. Table 1 provides the demographic and comorbidity characteristics of the ED study cohort with column percentages provided for each characteristic. In both 2010 and 2015, a higher proportion of patients with multiple ED visits were adults aged 18 to 24 years and 65 years and older, living in areas with lower income, living in rural locations, and having higher ADG comorbidities compared with those with a single ED visit. The 2015 ED cohort was older and had a higher proportion of comorbidities compared to the 2010 ED cohort.

**Table 1:** Demographic and comorbidity characteristics of emergency department cohorts in 2010 and 2015

	2010		2015	
	Patients with a Single ED visit (*N* = 19,659)	Patients with Multiple ED visits (*N* = 6950)	Patients with a Single ED visit (*N* = 37,182)	Patients with Multiple ED visits (*N* = 13,359)
Sex				
Female	11,150 (56.7%)	4107 (59.1%)*	20,670 (55.6%)	7599 (56.9%)
Male	8509 (43.3%)	2843 (40.9%)	16,512 (44.4%)	5760 (43.1%)
Age (Mean ± SD)	48.36 ± 18.08	48.83 ± 19.44	49.78 ± 19.05	52.24 ± 20.82*
Age Group (in years)				
18 to 24	2277 (11.6%)	941 (13.5%)*	3965 (10.7%)	1558 (11.7%)*
25 to 44	6203 (31.6%)	2071 (29.8%)	11,583 (31.2%)	3591 (26.9%)
45 to 64	7150 (36.4%)	2261 (32.5%)	12,574 (33.8%)	4026 (30.1%)
65 to 84	3644 (18.5%)	1482 (21.3%)	7704 (20.7%)	3352 (25.1%)
85+	385 (2.0%)	195 (2.8%)	1356 (3.6%)	832 (6.2%)
Neighbourhood Income Quintile				
1 – Lowest	3446 (17.5%)	1590 (22.9%)*	6913 (18.6%)	3251 (24.3%)*
2	3855 (19.6%)	1441 (20.7%)	7143 (19.2%)	2726 (20.4%)
3	4033 (20.5%)	1456 (20.9%)	7213 (19.4%)	2521 (18.9%)
4	4175 (21.2%)	1289 (18.5%)	7673 (20.6%)	2413 (18.1%)
5 - Highest	4081 (20.8%)	1138 (16.4%)	8062 (21.7%)	2376 (17.8%)
Rurality				
Major Urban	8370 (42.6%)	2028 (29.2%)*	20,343 (54.7%)	6035 (45.2%)*
Small Urban	8316 (42.3%)	3026 (43.5%)	12,213 (32.8%)	4612 (34.5%)
Rural	2973 (15.1%)	1896 (27.3%)	4626 (12.4%)	2712 (20.3%)
Comorbidity (ADG Groups)				
0–4 (lower)	10,630 (54.1%)	2423 (34.9%)*	19,499 (52.4%)	4311 (32.3%)*
5–9	8137 (41.4%)	3721 (53.5%)	15,630 (42.0%)	6933 (51.9%)
10+ (higher)	902 (4.6%)	806 (11.6%)	2053 (5.5%)	2115 (15.8%)
Chronic Disease Cohort				
Post AMI	390 (2.0%)	71 (1.0%)*	827 (2.2%)	119 (0.9%)*
Asthma	3095 (15.7%)	1040 (15.0%)	6212 (16.7%)	1845 (13.8%)*
CHF	457 (2.3%)	66 (0.9%)*	1211 (3.3%)	108 (0.8%)*
COPD	1721 (8.8%)	381 (5.5%)*	3833 (10.3%)	705 (5.3%)*
Diabetes	2251 (11.5%)	432 (6.2%)*	4860 (13.1%)	743 (5.6%)*
Hypertension	5524 (28.1%)	1233 (17.7%)*	10,812 (29.1%)	2178 (16.3%)*
Mental Health	14,524 (73.9%)	3956 (56.9%)*	28,428 (76.5%)	7219 (54.0%)*

*p < 0.001

*ADG* Adjusted Diagnostic Group, *Post AMI* Post acute myocardial infarction, *CHF* Congestive Heart Failure, *COPD* Chronic obstructive pulmonary disease

Table 2 describes the receipt of an ED note (and type of note) by a patient’s FP. FPs received an ED note from 10,471 (53.3%) patients and from 15,257 (41.0%) patients having a single ED visit in 2010 and in 2015, respectively. For patients with multiple ED visits, the patient’s FP received an ED note from 4044 (58.2%) patients in 2010 and from 6106 (45.7%) patients in 2015. In 2010, a higher proportion of ED notes received by FPs were ED consultation notes in comparison to 2015.

**Table 2:** The receipt of an emergency department note by the patient’s family physician

	2010		2015	
	Patients with a Single ED visit (*N* = 19,659)	Patients with Multiple ED visits (*N* = 6950)	Patients with a Single ED visit (*N* = 37,182)	Patients with Multiple ED visits (*N* = 13,359)
**Getting any ED note**	10,471 (53.3%)*	4044 (58.2%)	15,257 (41.0%)*	6106 (45.7%)
***ED Consultation Note only***	6896 (65.9%)*	2055 (50.8%)	7519 (49.3%)*	2218 (36.3%)
***ED Laboratory Results***	3225 (30.8%)*	1105 (27.3%)	6535 (42.8%)*	2175 (35.6%)
***Miscellaneous ED Notes***	350 (3.3%)*	884 (21.9%)	1203 (7.9%)*	1713 (28.1%)

*p < 0.001

Table 3 compares the characteristics of single and multiple ED visit patients whose FPs received or did not receive an ED note in 2015. For patients with either a single ED visit or multiple ED visits, FPs received an ED note from a higher proportion of patients who were older, were living in urban areas, and had moderate to high comorbidity compared to patients whose FP did not receive an ED note. A lower proportion of patient’s FP received an ED note were living in the lowest neighbourhood income quintile for single ED visits, but not for multiple ED visits. For patients with CHF, COPD, diabetes, hypertension and mental health conditions having a single ED visit only, a higher proportion of their FPs received an ED note.

**Table 3:** Comparison of ED patients whose FP did receive or did not receive an ED note in 2015

	Single ED Visit		Multiple ED Visits	
	FP did receive an ED note (*N* = 15,257)	FP did not receive an ED note (*N* = 21,925)	FP who did receive an ED note (*N* = 6106)	FP did not receive an ED note (*N* = 7253)
Sex				
Female	8624 (56.5%)	12,046 (54.9%)	3515 (57.6%)	4084 (56.3%)
Male	6633 (43.5%)	9879 (45.1%)	2591 (42.4%)	3169 (43.7%)
Age (Mean ± SD)	51.85 ± 19.18	48.34 ± 18.83*	53.68 ± 20.76	51.02 ± 20.79*
Age Group (in years)				
18 to 24	1394 (9.1%)	2571 (11.7%)*	635 (10.4%)	923 (12.7%)*
25 to 44	4291 (28.1%)	7292 (33.3%)	1542 (25.3%)	2049 (28.3%)
45 to 64	5276 (34.6%)	7298 (33.3%)	1874 (30.7%)	2152 (29.7%)
65 to 84	3604 (23.6%)	4100 (18.7%)	1627 (26.6%)	1725 (23.8%)
85+	692 (4.5%)	664 (3.0%)	428 (7.0%)	404 (5.6%)
Neighbourhood Income Quintile				
1 – Lowest	2561 (16.8%)	4352 (19.8%)*	1340 (21.9%)	1911 (26.3%)*
2	2989 (19.6%)	4154 (18.9%)	1253 (20.5%)	1473 (20.3%)
3	3092 (20.3%)	4121 (18.8%)	1191 (19.5%)	1330 (18.3%)
4	3331 (21.8%)	4342 (19.8%)	1199 (19.6%)	1214 (16.7%)
5 - Highest	3235 (21.2%)	4827 (22.0%)	1097 (18.0%)	1279 (17.6%)
Rurality				
Major Urban	7583 (49.7%)	12,760 (58.2%)*	2781 (45.5%)	3254 (44.9%)*
Small Urban	6614 (43.4%)	5599 (25.5%)	2598 (42.5%)	2014 (27.8%)
Rural	1060 (6.9%)	3566 (16.3%)	727 (11.9%)	1985 (27.4%)
ADG Groups				
0–4 (Lower)	7470 (48.9%)	12,029 (54.9%)*	1794 (29.4%)	2516 (34.7%)*
5–9	6858 (44.9%)	8772 (40.0%)	3356 (55.0%)	3577 (49.3%)
10+ (Higher)	929 (6.1%)	1124 (5.1%)	955 (15.6%)	1160 (16.0%)
Chronic Disease Cohorts				
Post AMI	378 (2.5%)	449 (2.0%)	60 (1.0%)	59 (0.8%)
Asthma	2541 (16.7%)	3671 (16.7%)	818 (13.4%)	1027 (14.2%)
CHF	608 (4.0%)	603 (2.8%)*	53 (0.9%)	55 (0.8%)
COPD	1738 (11.4%)	2095 (9.6%)*	330 (5.4%)	375 (5.2%)
Diabetes	2217 (14.5%)	2643 (12.1%)*	346 (5.7%)	397 (5.5%)
Hypertension	5043 (33.1%)	5769 (26.3%)*	1049 (17.2%)	1129 (15.6%)
Mental Health	11,858 (77.7%)	16,570 (75.6%)*	3340 (54.7%)	3879 (53.5%)

*p < 0.001

*ADG* Adjusted Diagnostic Group, *Post AMI* Post acute myocardial infarction, *CHF* Congestive Heart Failure, *COPD* Chronic obstructive pulmonary disease

An examination of patient factors associated with the receipt of an ED note by FPs are presented in Table 4. After adjusting for all other covariates, FPs were more likely to receive an ED note for patients not living in low income neighbourhoods. Compared to patients living in major urban areas, FPs were more likely to receive an ED note for patients living in small urban areas but less likely to receive them for patients living in rural areas. FPs were more likely to receive an ED note for patients having moderate comorbidity compared to the people with no or low comorbidity. Similar findings were found between patients having single versus multiple ED visits.

**Table 4:** Multivariate comparison of ED patients whose FP did receive or did not receive an ED note in 2015

	Single ED Visit		Multiple ED Visits	
	Odds Ratio (95%CI)	Pr > Chi-Square	Odds Ratio (95%CI)	Pr > Chi-Square
Age	1.01 (1.01, 1.01)	<.0001	1.01 (1.01, 1.01)	<.0001
Men vs Women	0.95 (0.91, 0.99)	0.0132	0.94 (0.87, 1.01)	0.0848
Neighbourhood Income Quintiles				
2 vs 1	1.22 (1.14, 1.31)	<.0001	1.31 (1.18, 1.46)	<.0001
3 vs 1	1.24 (1.16, 1.33)	<.0001	1.36 (1.22, 1.52)	<.0001
4 vs 1	1.19 (1.11, 1.27)	<.0001	1.36 (1.22, 1.52)	<.0001
5 vs 1	1.1 (1.03, 1.18)	0.0070	1.18 (1.05, 1.31)	0.0037
ADG Groups				
5 to 9 vs 0 to 4	1.11 (1.06, 1.16)	<.0001	1.2 (1.11, 1.3)	<.0001
10+ vs 0 to 4	1.06 (0.96, 1.17)	0.2538	0.98 (0.87, 1.1)	0.7556
Rurality				
Rural vs Major Urban	0.48 (0.44, 0.52)	<.0001	0.41 (0.37, 0.46)	<.0001
Small Urban vs Major Urban	1.98 (1.89, 2.07)	<.0001	1.53 (1.41, 1.65)	<.0001
Chronic Disease Cohorts				
Post AMI	0.98 (0.85, 1.14)	0.8257	1.06 (0.73, 1.55)	0.7582
Asthma	1 (0.94, 1.06)	0.9891	0.95 (0.86, 1.06)	0.3611
CHF	1.09 (0.96, 1.23)	0.2018	0.96 (0.64, 1.43)	0.8269
COPD	0.96 (0.89, 1.04)	0.3455	0.92 (0.78, 1.09)	0.3342
Diabetes	1.02 (0.96, 1.1)	0.4897	0.96 (0.82, 1.13)	0.6532
Hypertension	1.11 (1.05, 1.18)	0.0003	0.98 (0.87, 1.09)	0.6646
Mental Health	1.06 (1.01, 1.12)	0.0313	0.93 (0.87, 1)	0.0644

*ADG* Adjusted Diagnostic Group, *Post AMI* Post acute myocardial infarction, *CHF* Congestive Heart Failure, *COPD* Chronic obstructive pulmonary disease

## Discussion

In contrast to the receipt of a discharge note after a hospital admission, there are no established benchmarks for FPs receiving information about their patients’ ED visits. In our study, not quite half of patients visiting the ED had information provided to their FP. We used a broad definition of an ED note in this study. An ED note could include an encounter or consultation note, a notification that a patient was seen and/or discharged from the ED or the results of laboratory or radiological tests. We also credited multiple ED visits as having provided the FP a note if anyone of the ED visits had an associated note found in the FP’s EMR.

While EMR use for clinical care is similar between FPs and specialist physicians in Ontario [49], the proportion of Ontario ED physicians documenting their clinical encounters using EMRs is unknown. The implementation of EHRs in EDs continues to evolve in Ontario. EHR/EMR implementation and adoption poses unique challenges as an ED cannot shut down while testing a system and the impact of ED physician allocation and efficiencies are different than office-based practices [50]. A recent Ontario study found a reduction in ED physician efficiencies over time that did not recover to baseline after the implementation of an ED EHR [51]. However an important objective of the digital health strategy is to improved communications with providers across healthcare sectors. In our study we found a lower rate of communication from EDs to community FPs in 2015 compared to 2010. Benchmarks for communication with community providers need to be established and regularly measured as EDs adopt EHRs to ensure that information is truly received by community providers in a timely manner.

Whether a written note is FAXed to a community FP or an electronic note is directly sent into the FPs EMR, it is a necessary first step that the patient’s FP be identified when coming to the ED. One reason that FPs do not receive information is because the FP’s contact information held in EDs may be inaccurate or unknown. Similar to our study, a recent survey found over half of Canadian FPs (59% in Ontario) say they receive notification of an ED visit [30]. Indeed only 22% of Canadian FPs said they could communicate patient clinical summaries across health care sectors [16]. EHR technology is already imbedded in ED and primary care practices. However, improvements are needed with the integration of technologies that interface across different healthcare settings to improve communication between EDs and community FPs in a seamless manner, without increasing the workload on either busy provider.

Rural patients were less likely to have their FPs received an ED note. However, FPs practicing in rural areas may see their own patients in the ED and therefore may not send themselves an ED note [52]. These ED visits may be related to chronic disease management, rather than an acute condition. We found moderate comorbidity was associated with the receipt of ED notes, yet having a chronic condition did not. Our measure of comorbidity (ADGs) are case mix measures that combine health conditions to determine a person’s need for health services [39]. Patients having more health care services may be a trigger to send information to the FP. However, patients with a defined chronic condition may be stable with respect to that condition, or see the ED for another reason, and this does not prompt ED providers to send information to the community FP.

Our study included a large cohort of FPs from rural and urban regions in Ontario and practicing in academic and community settings. We used EMR and health administrative data, as opposed to survey data which is susceptible to recall bias. While our study cohort of FPs and their patients are similar to FPs practicing in Ontario, it does over represent rural FPs and Canadian trained FPs [34]. We did not examine the quality of the ED information received, nor the receipt of ED notes by any specific acute or chronic disease conditions. We did not examine telephone or other direct communication between ED providers and FPs and therefore may be underestimating communication about ED visits. Finally, we did not examine the influence of ED provider or ED characteristics (such as their level of EHR adoption) on the receipt of ED information by community FPs.

## Conclusions

FPs are more likely to get information after an ED visit for their older and sicker patients. However, FPs do not receive information from EDs for over half their patients. Electronic health record technologies, alongside the adoption of their use by ED providers need to improve the seamless transfer of information about the care provided in ED to FPs in the community.

## Data Availability

The dataset from this study is held securely in coded form at ICES. While legal data sharing agreements between ICES and data providers (e.g., healthcare organizations and government) prohibit ICES from making the dataset publicly available, access may be granted to those who meet pre-specified criteria for confidential access, available at www.ices.on.ca/DAS (email: das@ices.on.ca). The full dataset creation plan and underlying analytic code are available from the authors upon request, understanding that the computer programs may rely upon coding templates or macros that are unique to ICES and are therefore either inaccessible or may require modification.
